# Supplementary material for: Cloud BioLinux: pre-configured and on-demand bioinformatics computing for the genomics community
Source: BMC Bioinformatics. 2012 Mar 19;13:42. doi: 10.1186/1471-2105-13-42 (PMC3372431; doi:10.1186/1471-2105-13-42)
Supplement: Additional file 1 — Supplementary 1 Cloud BioLinux software documentation in the form of a mini, self-contained website. Users need to download and uncompress the .zip file, and open through a web browser the "index.html" file available on the main directory. (ZIP 1823 kb). [file 1471-2105-13-42-S1.ZIP › Cloud-BioLinux-Package-Documentation/docs/clobb.html]

Bio-Linux Software Documentation Pages

Back to search form

## clobb

|  |  |
| --- | --- |
| Name | clobb |
| Description | **CLOBB** (Cluster on the basis of BLAST similarity), is a clustering algorithm to identify and cluster ESTs.  **References:**  Making sense of EST sequences by CLOBBing them. John Parkinson, David B. Guiliano, and Mark Blaxter. Institute of Cell, Animal and Population Biology, University of Edinburgh, Edinburgh EH9 3JT, UK. BMC Bioinformatics. 2002; 3 (1): 31 |
| Homepage | http://nema.cap.ed.ac.uk/CLOBB/ |
| Remote Documentation | http://nema.cap.ed.ac.uk/CLOBB/CLOBB.POD |
